# Regulation of plant immunity via small RNA-mediated control of NLR expression

**DOI:** 10.1093/jxb/erad268

**Published:** 2023-07-14

**Authors:** Diego López-Márquez, Ángel Del-Espino, Javier Ruiz-Albert, Eduardo R Bejarano, Peter Brodersen, Carmen R Beuzón

**Affiliations:** Department of Biology, University of Copenhagen, Copenhagen N, DK-2200, Denmark; Instituto de Hortofruticultura Subtropical y Mediterránea ‘La Mayora’, Universidad de Málaga-Consejo Superior de Investigaciones Científicas (IHSM-UMA-CSIC), Depto. Biología Celular, Genética y Fisiología, Málaga, Spain; Instituto de Hortofruticultura Subtropical y Mediterránea ‘La Mayora’, Universidad de Málaga-Consejo Superior de Investigaciones Científicas (IHSM-UMA-CSIC), Depto. Biología Celular, Genética y Fisiología, Málaga, Spain; Instituto de Hortofruticultura Subtropical y Mediterránea ‘La Mayora’, Universidad de Málaga-Consejo Superior de Investigaciones Científicas (IHSM-UMA-CSIC), Depto. Biología Celular, Genética y Fisiología, Málaga, Spain; Department of Biology, University of Copenhagen, Copenhagen N, DK-2200, Denmark; Instituto de Hortofruticultura Subtropical y Mediterránea ‘La Mayora’, Universidad de Málaga-Consejo Superior de Investigaciones Científicas (IHSM-UMA-CSIC), Depto. Biología Celular, Genética y Fisiología, Málaga, Spain; CSIC, Spain

**Keywords:** Effector-triggered immunity, miRNA, NLR proteins, plant immunity, post-transcriptional gene silencing, *R* genes, RNAi, secondary siRNA

## Abstract

Plants use different receptors to detect potential pathogens: membrane-anchored pattern recognition receptors (PRRs) activated upon perception of pathogen-associated molecular patterns (PAMPs) that elicit pattern-triggered immunity (PTI); and intracellular nucleotide-binding leucine-rich repeat proteins (NLRs) activated by detection of pathogen-derived effectors, activating effector-triggered immunity (ETI). The interconnections between PTI and ETI responses have been increasingly reported. Elevated NLR levels may cause autoimmunity, with symptoms ranging from fitness cost to developmental arrest, sometimes combined with run-away cell death, making accurate control of NLR dosage key for plant survival. Small RNA-mediated gene regulation has emerged as a major mechanism of control of NLR dosage. Twenty-two nucleotide miRNAs with the unique ability to trigger secondary siRNA production from target transcripts are particularly prevalent in NLR regulation. They enhance repression of the primary NLR target, but also bring about repression of NLRs only complementary to secondary siRNAs. We summarize current knowledge on miRNAs and siRNAs in the regulation of NLR expression with an emphasis on 22 nt miRNAs and propose that miRNA and siRNA regulation of NLR levels provides additional links between PTI and NLR defense pathways to increase plant responsiveness against a broad spectrum of pathogens and control an efficient deployment of defenses.

## Teaser

miRNAs regulate plant immunity through repression of mRNAs encoding nucleotide binding-leucine-rich repeat intracellular receptors (NLRs), often triggering production of siRNAs that reinforce repression and direct silencing of additional NLRs.

## Introduction

**Fig. 1.**
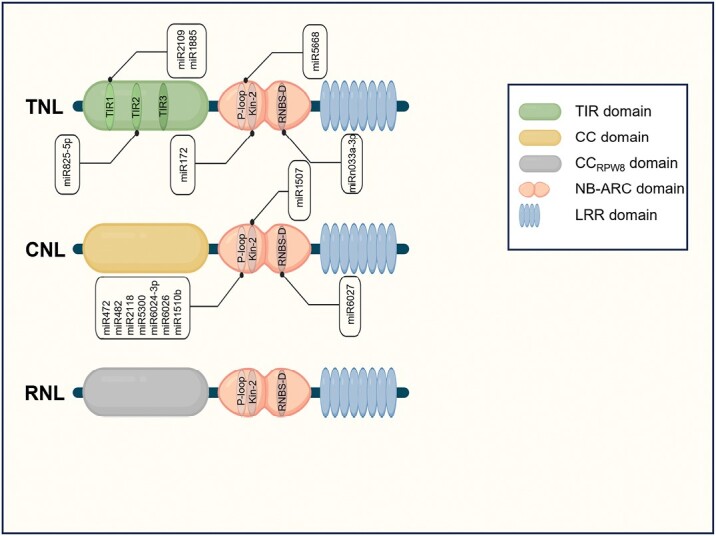
NLRs are targeted by miRNAs at highly conserved motifs. According to its N-terminal domain, the three main clades of plant NLRs are depicted. Conserved motifs and experimentally validated miRNAs are highlighted. TNL, Toll/interleukin-1 (TIR) type NLR; CNL, coiled-coil (CC) type NLR; RNL, RPW8-like CC-type NLR. The figure was created with BioRender.com.

Plants use cell surface and intracellular receptors to detect potential pathogens. Activation of membrane-anchored pattern recognition receptors (PRRs) is typically associated with perception of pathogen-associated molecular patterns (PAMPs) and elicits pattern-triggered immunity (PTI). Intracellular nucleotide-binding (NB) leucine-rich repeat (LRR) receptors (NLRs) detect pathogen-derived effectors, thereby activating effector-triggered immunity (ETI). Pathogen effectors act intracellularly to manipulate host processes and facilitate pathogen spread. The immune response triggered by the activation of NLRs, known as ETI, is fast and robust, and is often accompanied by activation of localized programmed cell death known as the hypersensitive response (HR) (Dodds and Rathjen, 2010; Cui *et al.*, 2015). Molecularly, NLRs display a multidomain structure that includes a conserved NB and an LRR domain accompanied in most cases by N-terminal domains that fall into one of three major classes (Meyers *et al.*, 2003; Cui *et al.*, 2015; Zhang *et al.*, 2017): Toll/interleukin-1 (TIR) domains (TNLs), coiled-coil (CC) domains (CNLs), and RPW8-like CC-type domains (RPW8-NLRs or RNLs) (Fig. 1) (Shao *et al*., 2016; Jubic *et al.*, 2019).

Genes encoding NLRs or, in rare cases, TIR-only or TIR-NB variants (Cesari, 2018), make up most of the *Resistance* (*R*) genes that mediate gene-for-gene resistance to pathogens carrying cognate effectors, in this case known as avirulence factors (Flor, 1971). NLRs may act as both sensors and signal transducers. Sometimes they act in pairs such that a sensor NLR is coupled to a so-called helper NLR that triggers signaling. Remarkably, such coupled sensor–helper NLRs are often encoded by tightly linked genes. The principle of coupled sensor and helper NLRs may be extended to become networks, where a large number of sensor NLRs require multiple helper NLRs, not always encoded within clusters in the genome, to mediate resistance against an assortment of effectors and pathogens (Adachi *et al.*, 2019). This increased complexity may reflect NLR evolution driven by the need to keep up with rapidly evolving pathogens (Adachi *et al.*, 2019).

Plant NLRs remain in an inactive form through intramolecular associations prior to effector recognition. Effector recognition by plant NLRs may take place through direct interaction with the effector, by recognition of effector-mediated modifications of a host target protein (guard model) or a protein that mimics its target protein (decoy model), or through direct modification by the effector of a decoy domain integrated within the NLR protein (Cesari, 2018). Activation upon recognition proceeds in a complicated process that involves nucleotide exchange, ATP binding, and chaperone-assisted conformational changes, ultimately resulting in multimerization (Ve *et al.*, 2015; Bentham *et al.*, 2018).

Recent structural and molecular studies on CNLs and TNLs have provided detailed insight into NLR multimerization and function. Upon effector activation of the CNL ZAR1, inactive monomers form a pentameric wheel-like complex termed a resistosome (Wang *et al.*, 2019a, b). This pentamerization triggers the release of the terminal α-helices of the CC domain, which go from being buried within the inactive monomer to projecting out of the resistosome plane to form a funnel-like structure required for cell death-inducing activity (Wang *et al.*, 2019a, b). The funnel inserts itself into the plasma membrane forming a pore or channel, potentially releasing host defense-potentiating molecules and/or promoting inward Ca^2+^ fluxes to drive intracellular signaling cascades (Wang *et al.*, 2019a, b; Bi *et al.*, 2021; Dongus and Parker, 2021). Key advances have also been made on plant TIR resistosomes: upon effector-induced TNL tetramerization (Ma *et al*., 2020; Martin *et al*., 2020), a holoenzyme is formed in which the TIR domains have an enzymatic activity that cleaves NAD^+^ into nicotinamide (Nam) (Essuman *et al*., 2018) and a variety of ADP-ribose (ADPR) isomers (Manik *et al.*, 2022), and related molecules (Horsefield *et al*., 2019; Wan *et al*., 2019; Huang *et al.*, 2022; Jia *et al.*, 2022; Leavitt *et al*., 2022), with signaling functions. Remarkably, TIRs may also polymerize to form fibers that have the distinct enzymatic activity of catalyzing hydrolysis of dsRNA or, *in vitro*, DNA, to form 2ʹ,3ʹ-cAMP or 2ʹ,3ʹ-cGMP (Essuman *et al.*, 2022; Yu *et al.*, 2022) with cell death-inducing function. Some of the molecules produced by TIR enzymatic activities are perceived by EDS1–PAD4 or EDS1–SAG101 heterodimeric nucleotide receptors that bind to and activate distinct helper NLRs upon nucleotide binding (Huang *et al.*, 2022; Jia *et al.*, 2022, 2023), while the biochemical function of others remains to be determined.

Activation of NLR-mediated pathways is under tight control. Many NLRs are involved in trade-offs between disease resistance and growth or response to abiotic stresses (Ariga *et al.*, 2017; Karasov *et al.*, 2017), and autoimmunity as a consequence of constitutive or misregulated activation can have deleterious effects, including severe growth suppression (Shirano *et al.*, 2002; Tian *et al.*, 2003; Korves and Bergelson, 2004; Palma *et al.*, 2010). NLR protein production undergoes regulation at the transcriptional, post-transcriptional, translational, and post-translational levels (Cheng *et al*., 2011; Gloggnitzer *et al*., 2014; Wu *et al*., 2017; Lai and Eulgem, 2018). Among these mechanisms, small RNA-guided repression at the post-transcriptional level has been found to be implemented across many different plant species to achieve appropriate NLR expression and regulation (Zhai *et al*., 2011; Li *et al*., 2012b; Shivaprasad *et al*., 2012; Fei *et al*., 2013). Small RNAs are at the core of RNAi phenomena that are of significant interest in plant–pathogen interactions because of the employment of effectors targeting RNAi pathways in many unrelated groups of pathogens, including viruses, bacteria, fungi, and oomycetes (Anandalakshmi *et al*., 1998; Voinnet *et al*., 1999; Navarro *et al*., 2008; Qiao *et al.*, 2013; Csorba *et al*., 2015; Yin *et al*., 2019). Two types of small RNAs, miRNAs and siRNAs, are involved in the post-transcriptional regulation of NLRs (Zhai *et al*., 2011; Li *et al*., 2012b; Shivaprasad *et al*., 2012; Fei *et al*., 2013), and the mechanistic underpinnings of this type of regulation and its possible significance in the broader perspective of plant–pathogen interactions will be the focus of the remainder of this review.

## Elements of the biogenesis and action of miRNAs

**Fig. 2.**
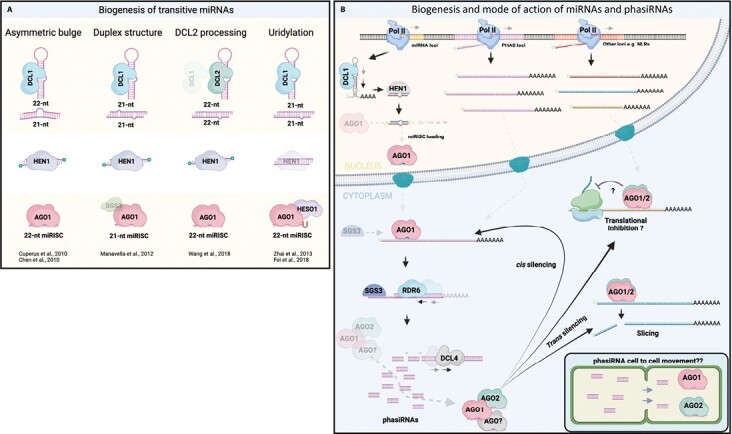
Biogenesis and mode of action of plant miRNAs and phasiRNAs. (A) Comparison of the models explaining the origin of transitivity inducer miRNAs. Three of the described examples are based on the action of a 22 nt miRNA, that is produced by (i) the presence of an asymmetric bulge in the pri-miRNA, (ii) by DCL2-dependent processing, or (iii) by HESO1-dependent mono-uridylation of a non-methylated 21 nt miRNA. Additionally, the structure of the miRNA/miRNA* also has been proposed to be key to trigger secondary siRNA biogenesis, even in the case of a 21/21 nt miRNA/miRNA* duplex. (B) Biogenesis and mode of action of miRNAs and phasiRNAs. The miRNA precursor (pri-miRNA) is transcribed by RNA polymerase II, folded into a hairpin structure, and chopped by DCL1 to render the miRNA/miRNA* duplex, that subsequently is methylated at the 3ʹ ends by HEN1. Afterwards, the duplex is loaded onto AGO1 and the miRNA* is ejected from the ribonucleoprotein complex to form a mature miRISC. Then, the complex is exported into the cytoplasm. As a first step of the phasiRNA biogenesis, the target mRNAs (e.g. NLRs) are sliced by a transitive inducer miRNA (see A), triggering the conversion of one of the cleavage fragments into a dsRNA by the action of RDR6, in a process assisted by SGS3 and SDE5 (not depicted). The dsRNA becomes a substrate of DCL4 to render secondary phased siRNAs (phasiRNAs) that load onto AGO1/AGO2 to regulate either the same mRNA (*cis* silencing) or other mRNAs (*trans* silencing), thus amplifying or reinforcing the silencing signal of the miRNA, respectively. PhasiRNA-dependent regulation may happen by slicing or translational inhibition (unknown). Notice that phasiRNA movement is also depicted (as proposed) in the model; however, whether phasiNLRs possess this property remains unknown. DCL, DICER-LIKE; HEN1, HUA ENHANCER 1; POLII, POLYMERASE II; AGO, ARGONAUTE; SGS3, SUPRESSOR OF GENE SILENCING 3; RDR6, RNA DEPENDENT RNA POLYMERASE 6. SDE5, SILENCING DEFECTIVE 5. The figure was created with BioRender.com.

Small silencing RNAs are ~21–24 nt single-stranded non-coding RNAs that explain the sequence specificity of RNAi by their base pairing to complementary RNA targets. These small RNAs always act in association with proteins of the ARGONAUTE (AGO) family (Vaucheret, 2008; Poulsen *et al*., 2013; Martin-Merchan *et al*., 2023). RNAi phenomena include both transcriptional and post-transcriptional repression, and, in plants, different classes of small RNAs can be defined according to their biogenesis and/or mode of action (Axtell, 2013; Bologna and Voinnet, 2014; Borges and Martienssen, 2015). miRNAs are 20–24 nt RNAs mainly involved in post-transcriptional regulation. They are key regulators of important plant processes, including plant development and stress responses (Voinnet, 2009). *MIRNA* loci are transcribed by RNA polymerase II, yielding poly(A)-tailed and m^7^G-capped primary miRNA transcripts (pri-miRNAs) containing a hairpin-like structure (Voinnet, 2009). Pri-miRNAs are processed into small RNA duplexes of ~21 nt by the dsRNA-directed RNAase DICER-LIKE1 (DCL1) (Park *et al*., 2002; Reinhart *et al*., 2002). In turn, the duplex consisting of miRNA and miRNA* strands undergoes protective ribose methylation at both 3ʹ ends, catalyzed by the methyl transferase HUA ENHANCER1 (HEN1) (Yu *et al*., 2005). The resulting methylated duplex is then loaded onto AGO1, the main ARGONAUTE protein operating in the miRNA pathway in plants (Vaucheret *et al*., 2004; Baumberger and Baulcombe, 2005). Subsequently, the miRNA* strand is ejected from AGO1, thus producing a mature miRNA-induced silencing complex (miRISC). The mature miRISC is the regulatory machine that exerts repression of target RNAs by endonucleolytic cleavage (slicing) or translational repression (Fig. 2) (Chen 2004; Vaucheret *et al*., 2004; Baumberger and Baulcombe, 2005; Qi *et al*., 2005; Brodersen *et al*., 2008).

## The special 22 nt miRNAs and triggering of the RNA-dependent RNA polymerase amplification module

Some plant pri-miRNAs contain asymmetric bulges. This may lead to the production of 22 nt miRNA/21 nt miRNA* duplexes such that an miRISC containing a 22 nt miRNA is formed. While this single nucleotide difference in small RNA size may seem minor, it is anything but that, since 22 nt miRNAs have a unique and powerful ability: triggering the conversion of their RNA targets into dsRNA by recruiting RNA-dependent RNA polymerase 6 (RDR6), in a process assisted by the proteins suppressor of gene silencing 3 (SGS3) and silencing defective 5 (SDE5) (Peragine *et al.*, 2004; Yoshikawa *et al*., 2005, 2021; Hernandez-Pinzón *et al.*, 2007; Chen *et al*., 2010; Cuperus *et al*., 2010; Arribas-Hernández *et al*., 2016; Sakurai *et al*., 2021). This dsRNA is cut into 21 nt sRNA duplexes by DCL4 (Xie *et al*., 2005) in a 21 nt phased pattern whose starting point is defined by the target RNA site where the 22 nt miRNA-guided slicing takes place. The resulting siRNAs are called phased secondary siRNAs (phasiRNAs), because of their phased pattern of accumulation and because their biogenesis depends on a primary trigger, in this case the 22 nt miRNA. The first phasiRNAs characterized were produced by miRNA-directed cleavage of long non-coding transcripts dedicated to production of secondary siRNAs that regulate mRNAs *in trans* like miRNAs, and, therefore, called *trans-*acting siRNAs (tasiRNAs) (Vazquez *et al*., 2004; Allen *et al*., 2005; Yoshikawa *et al*., 2005; Felippes and Weigel, 2009). Production of secondary siRNA does not require the slicer activity of AGO1 that is, however, essential for establishing a phased pattern (Arribas-Hernández *et al.*, 2016). In addition to precursor asymmetry, phasiRNA-triggering 22 nt miRNAs can be generated by mono-uridylation of miRNAs after DCL1 cleavage, or pri-miRNA processing by DCL2 that gives rise to small RNA duplexes 22 nt in size (Fig. 2) (Zhai *et al*., 2013; Tu *et al*., 2015; Fei *et al*., 2018; Wang *et al*., 2018). Nonetheless, an asymmetric duplex structure may confer additional properties on the resulting miRISC that enhance its ability to induce secondary siRNAs (Manavella *et al*., 2012).

PhasiRNA production may have at least three distinct outcomes. First, the silencing of the primary target RNA is amplified, a *cis* effect that is quantitative in nature. Second, additional target RNAs unrelated in sequence to the primary 22 nt trigger miRNA may be silenced by the phasiRNAs, a *trans* effect that is qualitative in nature (Fig. 2) (Allen *et al*., 2005; Yoshikawa *et al*., 2005; Zhai *et al*., 2011). Third, siRNAs produced via the RDR6-dependent amplification module show a markedly non-cell-autonomous mode of action, thus potentially extending the spatial boundaries of the domain of small RNA-mediated repression (Felippes *et al*., 2011).

## 22 nt miRNAs and phasiRNAs as widespread modulators of NLRs

The first reports of NLR regulation via miRNAs used 5ʹ-RNA ligase-mediated (RLM)-RACE to demonstrate that miR482 guides cleavage of NLR-encoding transcripts in *Populus trichocarpa*, while miR772 (renamed miR472) does so in Arabidopsis (Lu *et al*., 2005; Lu *et al.*, 2006). Later on, three reports (Zhai *et al*., 2011; Li *et al.*, 2012b; Shivaprasad *et al*., 2012) expanded significantly on these initial observations: (i) 22 nt miRNA-mediated NLR regulation through phasiRNA (phasiNLR) production is a recurrent mechanism across many plant species; (ii) although some trigger miRNAs are conserved across some species, the mechanism has evolved independently multiple times as evidenced by the use of distinct trigger miRNAs in different groups of species (Table 1); (iii) phasiRNAs bring about a second wave of NLR regulation (*cis* and *trans* modes); (iv) the trigger miRNA levels are modulated during pathogen–host interactions to allow for an increase in NLR expression; and (v) this modulation influences the outcome of the host–pathogen interaction. In the following, we expand on each of these important points, and discuss the use of the small RNA–NLR repression network to modulate pathogen detection and the plant responses through changes in NLR levels.

**Table 1.** Validated miRNAs that trigger phasiNLR biogenesis.

miRNA	Species^*a*^	miRNA size	Main miRNA targets^*b*^	Target site domain/motif	Evidence of miRNA activity^*c*^	PHAS loci	References
miR172	*S. lycopersicum/G. max*	21-nt	TNL	Kinase-2	PARE	Glyma18g14655	Seo *et al.* (2018)
miR472	*A. thaliana/P. trichocarpa*	22 nt	CNLs	P-loop	5ʹRACE and/or PARE	RPS5, RSG1-2, AT5G43740; PtPHA3-6	Lu *et al.* (2006); Boccara *et al.* (2014); Shuai *et al.* (2016); Su *et al.* (2018)
miR482	*P. trichocarpa/S. lycopersicum*	22 nt	TNL/CNL	P-loop	5ʹRACE and/or PARE	Several in each species	Lu *et al.* (2005); Shivaprasad *et al.* (2012); Jiang *et al.* (2018); Canto-Pastor *et al.* (2019)
miR825-5p	*A. thaliana*	22 nt	TNLs	TIR-2	PARE and reporter	MIST1	Howell *et al.* (2007); Chen *et al.* (2010); Niu *et al.* (2016); Nie *et al.* (2019); López-Márquez *et al.*, 2021
miR1507	*M. truncatula*	22 nt	CNL	Kinase-2	PARE	Medtr3g018980	Zhai *et al.* (2011)
miR1885	*B. rapa*	22 nt	TIR/TNL	TIR-1	5ʹRACE and reporter	BraTIR1, BraTNL1	Cui *et al.* (2020)
miR2109	*M. truncatula*	21 nt	TNL	TIR-1	PARE	Medtr1g044860	Zhai *et al.* (2011)
miR2118	*M. truncatula*	22 nt	TNL/CNL	P-loop	PARE	TAS5	Zhai *et al.* (2011); Canto-Pastor *et al.* (2019)
miR5300	*S. lycopersicum/S. tuberosum*	22 nt	CNL	P-loop	PARE	Tm2	Seo *et al.* (2018)
miR6019	*N. tabacum*	22 nt	TNL	TIR	5ʹRACE and/or PARE and/or reporter	N	Li *et al.* (2012b); Deng *et al.*, (2018)
miR6024-3p	*S. lycopersicum*	22 nt	CNL	P-loop	5ʹRACE	Rx1; NB5	Li *et al.* (2012b); Dey *et al.* (2022)
miR6026	*S. lycopersicum*	22 nt	CNL	P-loop	PARE	Tm2; Rpivnt1	Li *et al.* (2012b)
miR6027	*S. lycopersicum*	22 nt	CNL	RNBS-D	PARE	Sw5	Li *et al.* (2012b)
miR9863	*H. vulgare*	22 nt	CNL	ARC	5ʹRACE and reporter	MLA	Liu *et al.* (2014)
miR-n033a-3p	*Capsicum* spp./*S. tuberosum*	22 nt	TNL/CNL	RNBS-D	PARE	CA06g00990	Seo *et al.* (2018)
miR1510b	*G. max*	22 nt	CNL/TNL	P-loop	PARE	Glyma04g39740	Arikit *et al.* (2014); Cui *et al.* (2017)
miR3710	*P. abies*	21 nt	TNL	NB/LRR	PARE	MA_9397599g0010	Xia *et al.* (2015)
miR951	*P. abies*	22 nt	TNL	TIR	PARE	MA_117084g0010	Xia *et al.* (2015)
miR946	*P. abies*	22 nt	NLR	NB	PARE	MA_10430203g0010	Xia *et al.* (2015)
miR11476	*P. abies*	22 nt	NLR	NB	PARE	MA_10394670g0010	Xia *et al.* (2015)
miR11482	*P. abies*	22 nt	NLR	NB	PARE	MA_10062820g0010	Xia *et al.* (2015)
miR950	*P. abies*	22 nt	TNL	TIR	PARE	MA_8760260g0010	Xia *et al.* (2015)
miR1311	*P. abies*	22 nt	NLR	NB	PARE	MA_10435336g0010 (SUMM2)	Xia *et al.* (2015)
miR1312b	*P. abies*	22 nt	TNL	TIR	PARE	MA_10432475g0020	Xia *et al.* (2015)
miR11546	*P. abies*	22 nt	NLR	NB	PARE	MA_5784027g0010	Xia *et al.* (2015)
miR3697	*P. abies*	22 nt	TNL	TIR	PARE	MA_286751g0010	Xia *et al.* (2015)
miR3701a	*P. abies*	22 nt	NLR	NB	PARE	MA_460348g0010 (RPS5-like)	Xia *et al.* (2015)
miR3701b	*P. abies*	22 nt	NLR	TIR	PARE	MA_10316872g0010	Xia *et al.* (2015)
miR3709b	*P. abies*	22 nt	RNL	LRR	PARE	MA_10429865g0020 (RPP13)	Xia *et al.* (2015)
miR11506	*P. abies*	22 nt	TNL	TIR	PARE	MA_57122g0010 (TMV resistance N-like)	Xia *et al.* (2015)
miR11519	*P. abies*	22 nt	TNL	TIR	PARE	MA_395749g0010	Xia *et al.* (2015)
miR11532	*P. abies*	22 nt	TNL	TIR	PARE	MA_10436946g0010	Xia *et al.* (2015)
miR11511	*P. abies*	22 nt	TNL	TIR	PARE	MA_99743g0020	Xia *et al.* (2015)
miR11523	*P. abies*	22 nt	NLR	NB	PARE	MA_480656g0010	Xia *et al.* (2015)
miR11528	*P. abies*	22 nt	NLR	NB	PARE	MA_8555673g0010 (RPS2-like)	Xia *et al.* (2015)
miR5376	*G. max*	22 nt	CNL	CC	PARE	Glyma15g37320	Zhao *et al.* (2015)
miR5668	*G. max*	21 nt	TNL	Outside of P-loop	PARE	Glyma16g24920	Zhao *et al.* (2015)
miR5041	*G. max*	21 nt	CNL	CC	PARE	Glyma05g09440	Zhao *et al.* (2015)
miR1524	*G. max*	21 nt	RNL	–	PARE	Glyma03g04300	Zhao *et al.* (2015)
miR5669	*G. max*	22 nt	TNL	–	PARE	Glyma08g41270	Zhao *et al.* (2015)/Zhai *et al*. (2011)
miR5671	*G. max*	22 nt	TNL	–	PARE	Glyma14g23930	Zhao *et al.* (2015)/Zhai *et al*. (2011)
miR1536	*G. max*	21 nt	RNL	–	PARE	Glyma15g37820	Zhao *et al.* (2015)/Zhai *et al*. (2011)
miR1514	*G. max*	21 nt	TNL	–	PARE	Glyma16g03780	Zhao *et al.* (2015)/Zhai *et al*. (2011)
miR393	*G. max*	22 nt	TNL	–	PARE	Glyma16g10340	Zhao *et al.* (2015)/Zhai *et al*. (2011)
miR5767	*G. max*	21 nt	TNL	–	PARE	Glyma16g27560	Zhao *et al.* (2015)/Zhai *et al*. (2011)
miR5042	*G. max*	22 nt	RNL	–	PARE	Glyma18g51960	Zhao *et al.* (2015)/Zhai *et al*. (2011)
miR5163b-3p	*B. distachyon*	22 nt	RNL	LRR	PARE	Bradi4g10030	Zhang *et al.* (2016)

^*a*^ The species in which the activity of the miRNA was first validated and/or the phasiNLR biogenesis first detected. Note that the miRNA may be present in other species.

^*b*^ The main subclade of NLRs targeted by a given miRNA.

^*c*^ The methodology used to detect the miRNA activity over the target NLR (see Box 1).

## Evolution of miRNA-mediated NLR regulation

Phylogenetic analysis indicates that NLR emergence dates back to the common ancestor of the whole green lineage, followed by a rapid divergence into the TNL, CNL, and RNL subclades (Shao *et al*., 2019). The evolution of these receptors is highly dynamic and thoroughly influenced (and re-shaped) by pathogen–host interactions on an evolutionary time scale (Jacob *et al*., 2013; Borrelli *et al*., 2018; Tamborski and Krasileva, 2020). Accordingly, NLRs are polymorphic at the population level, and they define the repertoire of effectors that a given individual can detect (Kuang *et al*., 2004; Zhang *et al*., 2016). As with other gene families, a positive correlation exists between the number of *NLR* genes and the total number of genes in a plant genome (Wang *et al*., 2011). Even though *NLR* genes are the largest gene family giving rise to phasiRNAs (Fei *et al*., 2013; Liu *et al*., 2020), miRNA-based NLR regulation can be only traced back to the emergence of gymnosperms (Yue *et al*., 2012; Xia *et al*., 2015; Zhang *et al*., 2016), and phasiRNA biogenesis from NLRs seems to be more prevalent in eudicots than in monocots (Liu *et al*., 2020). Strikingly, a high degree of variability exists between the numbers of miRNA–NLR–phasiRNA networks within different species, which range from two miRNAs and a few NLRs triggering the production of phasiRNAs in *Arabidopsis thaliana*, to ~19 miRNAs and >750 NLRs doing so in *Picea abies* (Xia *et al*., 2015). The expansion of plant NLRomes and the high variability of NLR gene repertoires are governed by intricate processes of duplication, recombination, and genomic rearrangements (Friedman and Baker, 2007; Borrelli *et al*., 2018). These same processes are also believed to underpin the emergence of NLR-targeting miRNAs (Zhang *et al*., 2016), probably initially as inverted repeats of parts of NLR-encoding genes as suggested for emergence of *MIRNA* loci more generally (Allen *et al.*, 2004). The positive correlation between the size of a given NLR-gene family and the number of miRNAs that target it constitutes evidence for this proposition (González *et al.*, 2015; De Vries *et al*., 2015; Zhang e*t al*., 2016). Many miRNAs target NLRs at highly conserved motifs such as TIR2, P-loop, or Kinase-2, ensuring their activity over a substantial number of NLRs (Fig. 1; Table 1) (Zhai *et al*., 2011; Li *et al*., 2012b; Shivaprasad *et al*., 2012; Fei *et al*., 2013). The evolutionary history of these miRNAs may reflect some traces of convergent evolution, with the P-loop motif as the most represented target sequence within NLR transcripts (Zhang *et al*., 2016). Indeed, the P-loop is a target of the ancient miR482/miR2118 superfamily but is also targeted by many other miRNA families that emerged in specific plant lineages (e.g. miR6024 in *Solanaceae* or miR1510 in *Fabaceae*) (Zhang *et al*., 2016). Interestingly, a large-scale analysis of the P-loop motif and miR482 sequences over 400 million years of evolution indicated that the diversification of NLRs drives the evolution of the miR482/2118 superfamily. Whether selection for miRNA–target pairing imposes any constraints in NLR diversification is unclear (Zhang *et al*., 2016). In any case, targeting of conserved sequences coding for motifs relevant for protein function, as in many miRNA–NLR–mRNA interactions (Table 1), provides an evolutionary link between protein function and miRNA-mediated regulation.

## Mode of action of NLR-targeting miRNAs and phasiRNAs

Despite the occurrence of miRNA-induced phasiNLR production in many plant species, only a few examples of their mode of action have been described in detail. High-throughput sequencing-based parallel analysis of RNA ends (PARE, Box 1) in *Medicago truncatula* provided the first evidence of phasiNLR activity, as clear signatures of their activity by generation of cleaved fragments of both primary targeted NLR transcripts (*cis* silencing) and secondary NLR targets (*trans* silencing) were found (see Zhai *et al*., 2011). Using RLM-RACE (Box 1), slicing activity guided by a miR482-triggered phasiRNA derived from the NLR-encoding *LRR1* gene was also demonstrated in *Solanaceae* (Shivaprasad *et al*., 2012). Remarkably, this functional phasiNLR, which is abundant in *S. lycopersicum*, is lost in *S. pennellii*, illustrating substantial natural variation in the system. Slicing activity of phasiNLR on secondary targets has also been shown in tobacco (Deng *et al.*, 2018). In Arabidopsis, miR472-RDR6-dependent siRNAs have been proposed to regulate NLR abundance and the immune response against *P. syringae* (Boccara *et al*., 2014). However, experimental evidence supporting this notion is based on the analysis of the *rdr6-15* null mutant, which also exhibits altered auxin responses (Allen *et al*., 2005; Williams *et al*., 2005; Fahlgren *et al*., 2006). More recently, analysis of Arabidopsis PARE libraries provided evidence of the activity of several phasiNLRs, triggered by miR825-5p on its main target, the TNL-encoding *MIST1* transcript, on both primary (*cis*-silencing) and secondary (*trans*) NLR target genes (López-Márquez *et al*., 2021). Additionally, a *Brassica napus* phasiRNA (phasiR130-4) whose production is triggered by the miR1885–*BraTIR1* mRNA interaction, mediates *trans* silencing of *BraCP24* mRNA, at least when the two are transiently co-expressed in *Nicotiana benthamiana* leaves (Cui *et al.*, 2020). This example is somewhat unusual because *BraCP24* is not an NLR-encoding gene and is involved in development, not defense, and because phasiRNA130-4 arises from the mRNA of a TIR-only protein. Nonetheless, this case is of relevance since it is one of the very few examples in which changes in the trigger miRNA levels have been shown to significantly impact endogenous levels of a secondary target (*BraCP24*) mRNA (Cui *et al.*, 2020). In fact, while PARE and/or RLM-RACE analysis have demonstrated phasiNLR-mediated cleavage of both *cis* and *trans* NLR target genes (Zhai *et al*., 2011; López-Márquez *et al*., 2021), limited mRNA-seq evidence supports the impact of changes in miRNA/phasiNLR levels on secondary target mRNA levels. For example, changes in miR482/2118 levels (Box 2) in tomato significantly impact disease resistance, but this clear physiological effect is hardly explained by the rather small changes detected in NLR mRNA accumulation by RNA-seq (Canto-Pastor *et al.*, 2019). The authors proposed that this discordance between the impact on target mRNA accumulation and on defense activation supports the notion of a mixed mode of action for miRNAs and phasiNLRs, with part of the silencing activity being carried out through translational repression. In *N. benthamiana*, translational repression of NLR mRNAs has been experimentally supported by transient expression of *Triticeae*-specific miR9863a and tomato miR482f followed by mRNA and protein analysis (Liu *et al*., 2014; Ouyang *et al*., 2014). siRNAs have been shown to regulate mRNA targets through translational repression in other targets (Brodersen *et al*., 2008; Wu *et al*., 2020), a phenomenon that, if demonstrated for the regulation of NLRs, would add to the complexity of this regulation and could potentially constitute an important layer of phasiNLR-dependent modulation. Alternatively, considering the typically large number of primary and secondary target NLRs under miRNA regulation, the cumulative effect of small decreases in the mRNA levels of each individual NLR because of miRNA- and/or phasiNLRs-mediated degradation might eventually amount to a significant impact on immunity, in a context that may be hard to detect by RNA-seq experiments (López-Márquez *et al.*, 2021).

Box 1. **Experimental validation of miRNA/phasiRNA targets**

**5ʹRLM-RACE or RNA ligase-mediated rapid amplification of cDNA ends**. Consists of a modified RACE protocol with adapter ligation directly to 5ʹ-phosphorylated RNA ends, that constitutes the product of RISC slicer activity over target RNAs. This methodology detects the cleavage of a specific RNA targets with nucleotide resolution.

**PARE or parallel analysis of RNA ends (degradome sequencing).** A modified version of the 5ʹRLM-RACE technology in which the ligated products are subjected to high-throughput sequencing. This approach is useful to detect cleavage of RNA targets in a transcriptome-wide manner.

**miRNA (or phasiRNA) reporter activity**. Analysis of the expression of a modified version of a reporter gene (e.g. *GFP* or *GUS*) that harbors the miRNA target site of interest (which can be within the context of the genomic sequence or the CDS, or without) and thus is under miRNA-mediated regulation. When using this methodology, it is important to analyze in parallel a modified version of this construct carrying mutations that prevent miRNA (or phasiRNA) pairing without altering the protein sequence, as a control (non-regulated version of the reporter). This technique allows detection of both mRNA cleavage and translational inhibition, the latter not detected by techniques such as 5ʹRLM-RACE or PARE.

Box 2. **Methods to generate plant genotypes with altered levels of miRNA**

**Reducing miRNA levels.** Target Mimics (MIMs), Short Tandem Target Mimics (STTMs), and Central mismatches Molecular Sponges (cmSPs) technologies constitute the toolkit used in plant research to reduce mature miRNA levels. These three techniques make use of a non-coding RNA that harbors 1–15 non-cleavable altered miRNA target sites (mismatches or bulges) promoting miRNA knockdown, through a process known as target-directed miRNA degradation (TDMD). The main differences between the three methodologies are the sequence/structure of the non-coding RNA, the mismatch region, and the number of miRNA target sites per molecule of RNA decoy.

**Increasing miRNA levels**. Overexpression (e.g. 35S promoter) of the genomic sequence of the miRNA precursor (pri-miRNA) or use of artificial miRNAs (amiRNAs) in which the precursor of a known miRNA (e.g. miR319) is modified to harbor and render the mature sequence of the miRNA of interest.

The mode of action of small RNAs is governed by its AGO partner (Martin-Merchan *et al*., 2023). Analyses of AGO-bound small RNA fractions have shown that different members of the AGO family may be involved in NLR regulation, with a substantial amount of TNL-derived phasiRNAs bound to AGO1 and AGO2 in Arabidopsis (López-Márquez et al., 2021). Clearly, differences in AGO activity domains, their subcellular localizations, molecular functions, or even their expression profiles under stress conditions (e.g. pathogen attack) could influence the activity of phasiNLRs. It is noteworthy that the number of encoded AGO proteins is highly variable in plants (Martin-Merchan *et al*., 2023), a phenomenon that may contribute to functional specialization. Thus, future research in other plant species encoding a larger number of AGO proteins and phasiNLR loci (e.g. soybean) may further elucidate how AGO–phasiNLR networks operate.

Movement of siRNAs affects silencing effectiveness and has broad implications for plant development and immunity (Chitwood *et al.*, 2009; Shine *et al*., 2022). While many miRNAs tend to act within a short distance of their biogenesis site (10–15 cells), siRNAs generated through RDR6-dependent amplification are more prone to move between cells, contributing to spread of the silencing signal (Felippes *et al.*, 2011). Therefore, it is tempting to speculate that phasiNLRs may act to expand the domain of NLR repression beyond the expression domain of the trigger miRNA. However, this aspect of phasiNLR function remains to be experimentally explored.

## miRNA/phasiNLR regulation in response to pathogen detection

**Fig. 3.**
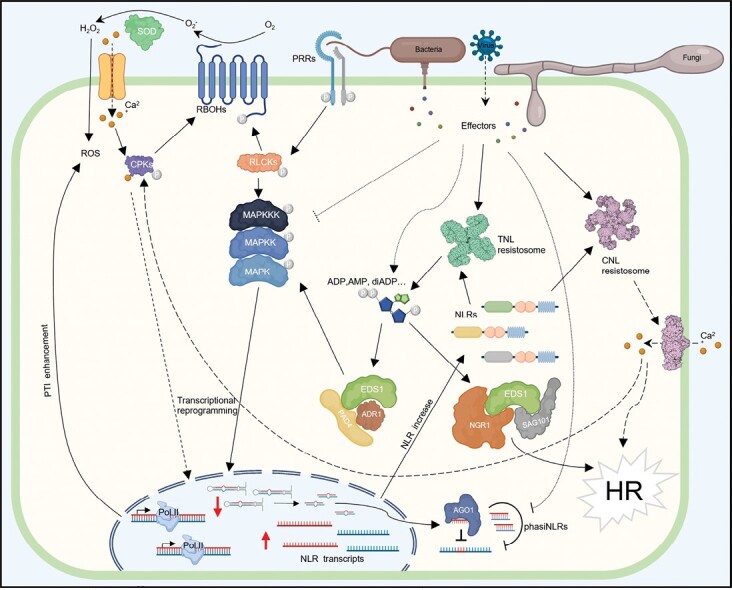
Overview of miRNA/phasiNLR regulation in relation to PRR- and NLR-mediated immunity. Schematic view of PRR- and NLR-mediated defense signaling and their connections. Solid arrows represent known defense signaling events. Dashed arrows show signaling processes not described at the molecular level. PRRs are modeled after the FLS2 receptor of bacterial flagellin and its co-receptor BAK1. The main components contributing to extracellular ROS signaling (RBOH and calcium channels) are depicted. RLCK, CPK, and MAPK cascades are represented generically. Effector activation of NLRs is shown with solid arrows while effector activities related to NLR-mediated PTI enhancement or silencing are indicated as dotted arrows. The figure was created with BioRender.com.

Intracellular NLR receptors have been considered for a long time as canonical elements of ETI, whereas membrane-anchored pattern recognition receptors (PRRs), activated upon perception of PAMPs such as bacterial flagellin, are canonical elements of PTI (Jones and Dangl, 2006). However, there is increasing evidence of considerable crosstalk and synergy between PRR-dependent (PTI) and NLR-dependent defense responses (ETI) (Fig. 3) (Hatsugai *et al*., 2017; Kadota *et al*., 2018; Lapin *et al*., 2020; [Bibr CIT0110][Bibr CIT0100]; Yuan *et al.*, 2021b). Activation of PRR and NLRs is triggered by ligands in different subcellular locations, but signals initiated from PRR and NLR converge upon common signaling elements (Lu and Tsuda, 2021). Additionally, a full NLR-dependent (ETI) response in Arabidopsis requires PRR-dependent activation (PTI) of some downstream components, such as membrane-bound NADPH oxidases or MAP kinases (Dongus and Parker, 2021; Ngou *et al*. 2021; Yuan *et al.*, 2021b). Furthermore, a subset of PRRs establishes PTI defense responses with the contribution of signaling components traditionally associated with NLR-activated signaling, such as the EDS1–PAD4 module (Pruitt *et al*., 2021; Tian *et al*., 2021; Feehan *et al*., 2023). This interplay between PRR- and NLR-mediated immune signaling has many implications for the analysis and characterization of plant–pathogen interactions and for our understanding of the role of miRNA/phasiNLR post-transcriptional regulation of NLRs in the plant responses against different biotic stresses. One mechanism by which activation of PRR signaling has been proposed to potentiate NLR-mediated defenses is through the regulation of gene expression (Ngou *et al.*, 2022). Transcriptional activation of multiple NLR genes has been shown to take place following PRR activation (Bjornson and Zipfel, 2021; Tian *et al.*, 2021). Although the mechanisms behind this link are not yet fully established, mitogen-activated protein kinase (MAPK) cascades and transcription factors (TFs) are likely to be involved (Ngou *et al.*, 2022). Alleviation of miRNA/phasiTNL silencing of NLR genes in response to PRR activation would contribute to establish such a connection.

Several reports have shown that miRNA-NLR/phasiNLR levels respond to pathogen sensing. The levels of the mature 22 nt miR472 are reduced after treatments with flg22 (poplar and Arabidopsis) or fungal PAMPs (Arabidopsis) (Li *et al*., 2010; Boccara *et al*., 2014; Su *et al*., 2018; Vasseur *et al.*, 2022, Preprint). Treatments with flg22 also lead to reduced levels of both the precursor and the mature miR825-5p in Arabidopsis (López-Márquez *et al.*, 2021). Thus, PRR signaling alone is sufficient to trigger changes in the levels of precursors and/or mature levels of miRNA in different plant species. The mechanism linking PRR activation to down-regulation of the levels of precursor and mature miRNAs involves transcriptional repression (Su *et al*., 2018; López-Márquez *et al.*, 2021), but remains largely unexplored. Repression may also take place at the level of mature miRNA and phasiNLR production, since *RDR6* and *AGO1* mRNA levels have been shown to rapidly decrease in Arabidopsis leaves and seedlings upon flg22 treatment (Boccara *et al.*, 2014). Whether NLR signaling alone could also trigger changes in the levels of precursors and/or mature levels of miRNA/phasiNLRs has not been investigated. Investigation of such a scenario would require analyses of miRNA/phasiNLR levels in transgenic plants expressing ETI-triggering effectors, since the use of bacterial delivery systems or *Agrobacterium*-mediated transient expression will produce confounding effects due to PRR activation. Thus, a model arises where NLR levels are kept low in the absence of the pathogen partly owing to miRNA/phasiNLR-mediated gene silencing, and these levels increase in response to pathogen-induced PRR-mediated signaling through both transcriptional activation of NLR-coding genes (Bjornson and Zipfel, 2021; Tian *et al.*, 2021) and alleviation of miRNA/phasiNLR repression (Fig. 3). In this model, miRNA/phasiNLR regulation would constitute an additional link between PRR- and NLR-mediated responses, with PRR-mediated pathogen perception increasing NLR protein levels.

## Role of miRNA/phasiNLR regulation in immunity

It is now clear that pathogen detection affects the levels of trigger miRNAs, but how does miRNA/phasiNLR regulation impact the outcome of the pathogen–host interaction? Most reports characterizing the impact of miRNA/phasiNLR regulation on plant immunity have shown that changes in the levels of these miRNAs (Box 2) alter resistance against virulent pathogens (i.e. that either successfully suppress ETI or trigger weak or no ETI) (Li *et al.*, 2012b; Boccara *et al.*, 2014; Canto-Pastor *et al*., 2019; López-Márquez *et al.*, 2021; Vasseur *et al.*, 2022, Preprint). In this context, alleviation of miRNA/phasiNLR repression leading to NLR increases could contribute to increase resistance by enhancing either PRR-activated responses or the level of ETI. Changes in the levels of some of these miRNAs (e.g. miR825-5p or miR472) specifically alter elicitation of plant immune responses by flg22, such as reactive oxygen species (ROS) production, callose deposition, or MAPK activation (Boccara *et al.*, 2014; Su *et al*., 2018; López-Márquez *et al.*, 2021). Thus, the NLRs regulated by these miRNA/phasiNLR modules are likely to be relevant for PRR-mediated immunity in the absence of specific NLR activation. Tian *et al.* (2021) showed that up-regulation of multiple TNLs boosts the immune responses against a *P. syringae* DC3000 *hrcC* mutant (which fails to introduce effectors into the host cell and therefore does not induce ETI) in Arabidopsis. The authors proposed that increases in the dosage of so many TIR genes could lead to an increased production of small molecules with defense signaling activities even in the absence of effector detection, assuming a certain degree of NLR misfiring, thus activating downstream TIR signaling pathways to a level sufficient to reinforce PTI responses without triggering cell death (Fig. 3). Nonetheless, given the increasing number of molecular links established between PRR and NLR immune signaling, several options are open to explain how increased levels of multiple NLR genes, many uncharacterized, can contribute to reinforce PRR-mediated immunity. For instance, a subset of the many uncharacterized miRNA/phasiNLR-regulated NLRs might act as helpers activated by EDS1–PAD4 complexes involved in PRR-mediated signaling, thus influencing the PTI response.

In Arabidopsis, the NLR-coding *RPS5* gene is a target for miR472-mediated silencing (Boccara *et al.*, 2014). RPS5 mediates ETI and HR activation in response to the bacterial effector AvrPphB (also known as HopAR1) (Simonich and Innes, 1995; Ade *et al*. 2007). Growth of a *P. syringae* DC3000 derivative expressing AvrPphB, which is usually limited by AvrPphB-triggered RPS5-dependent ETI, was significantly increased in plants overexpressing miR472, in keeping with a significant decrease of *RPS5* transcript levels. Since AvrPphB was delivered from a bacterial pathogen in this experimental setting, PRR activation may have occurred concomitantly with RPS5 activation. Such PRR activation is, however, likely to be residual because of its suppression by pathogen effectors. Interestingly, the authors did not detect any significant changes in bacterial growth of *P. syringae* DC3000 expressing the effector AvrRpt2, whose activity is detected by CNL RPS2 that is not a target of miR472. This observation supports the notion that the miR472 effect on signaling mediated by RPS5 activation was specific (Boccara *et al.*, 2014). In another study, mRNA accumulation of the defense marker gene *PR1* in response to a *P. syringae* DC3000 derivative expressing AvrRpt2 was significantly increased in *MIR825-*silenced Arabidopsis plants. RPS2 is not a known target for miR825/phasiNLR-mediated silencing (López-Márquez *et al.*, 2021). In this case, RPS2 may have been induced indirectly as a consequence of the alleviation of miR825-5p suppression on primary and secondary targets. Additionally, in *N. benthamiana, Agrobacterium*-mediated transient expression of miRNAs miR6019 and miR6020, which target transcripts of the NLR-coding gene *N*, attenuates *N*-mediated resistance to tomato mosaic virus (TMV) (Li *et al.*, 2012b).

Both miR472 and miR825-5p have been linked to the activation of the induced systemic response (ISR) in Arabidopsis (Niu *et al*., 2016; Nie *et al*., 2019; Jiang *et al*., 2020). The ISR is triggered by beneficial microbes and results in the activation of defense responses in local and systemic tissues that lead to a primed state where the plant can mount a faster and stronger cellular response upon pathogen attack (Conrath *et al.*, 2002). Levels of miR472 and miR825-5p decrease when ISR is activated (i.e. pre-treatment with *Bacillus cereus*) prior to infection with either *P. syringae* or *B. cinerea*, although this decrease is only statistically significant if pathogen infection follows ISR activation (i.e. treatment with *B. cereus* followed by *P. syringae* or *B. cinerea* infection) (Niu *et al*., 2016; Nie *et al*., 2019; Jiang *et al*., 2020). Whether changes in the levels of these miRNAs impact the ability of Arabidopsis to establish an ISR remains to be established.

## Why is miRNA/phasiRNA regulation of NLRs so prevalent?

NLR regulation by miRNAs, often involving 22 nt miRNAs and phasiNLR production, is widespread among different plant lineages (Table 1; Zhang *et al*., 2016). This recurrent pattern supports the importance of both regulatory nodes and begs the question of why NLR regulation through this combination of small RNA activities is so important. In the closing paragraphs, we discuss different possible explanations, none of them mutually exclusive, for this interesting question.

### Leveraging growth/defense dichotomy

Appropriate homeostasis of NLR activity is critical for plant performance (Li *et al.*, 2015). This condition is typically achieved by low-level expression under favorable growth conditions, and up-regulation in response to pathogen perception (Xiao *et al.*, 2003; Zipfel *et al.*, 2004). Indeed, effective activation of NLR-mediated defense signaling requires NLR protein levels to be above a certain threshold (Bieri *et al.*, 2004; Holt *et al.*, 2005). However, exacerbated NLR levels can cause autoimmune defects that include plant growth retardation, yield penalties, and, sometimes, necrosis (Mackey *et al.*, 2003; Xiao *et al.*, 2003). A simple, perhaps too simple, explanation for the importance of miRNA/phasiNLR down-regulation of NLR protein production in the absence of a pathogen is to achieve finely tuned NLR expression to reduce potential NLR-associated fitness losses (Li *et al*., 2012b; Shivaprasad *et al*., 2012; Fei *et al*., 2013; Boccara *et al*., 2014). This idea has been investigated by measuring the impact on plant fitness of elimination or exacerbation of miRNA/phasiNLR regulation. In Arabidopsis, overexpression of miR472 does cause a growth advantage in unchallenged conditions, although this advantage is small and without major effects on plant development. Conversely, knockdown and knockout of miR472 cause small growth delays (Jiang *et al*., 2020). An independent and more detailed study confirmed these results and showed that reduced levels of miR472 affected rosette growth, but not the age at bolting (Vasseur *et al*., 2022, Preprint). Also, in Arabidopsis, knockdown of both miR825-5p and miR825-3p, the two functional miRNAs generated from the same precursor, reduced plant growth, although in this case overexpression of both using artificial miRNAs did not have any impact (Niu *et al*., 2016). It is possible that stronger fitness costs could be associated with the loss of miRNA/phasiNLR regulation in other species where it is more prevalent (Zhang *et al*., 2016). However, knockdown of miR482 in tomato was not associated with gross effects on growth or development (Canto-Pastor *et al.*, 2019). In conclusion, the available evidence suggests that the miRNA/phasiNLR system is relevant for plant fitness, although the effects of its manipulation are relatively minor in unchallenged laboratory settings. In this model, the combined 22 nt trigger miRNA/phasiNLR activity could perhaps be associated with a larger reduction of fitness costs than simple repression by 21 nt miRNAs. In any case, these studies have analyzed the potential impact on fitness of the loss of miRNA/phasiNLR regulation in optimal growth conditions. Stronger impacts on plant growth are expected to be associated with lower levels of these miRNAs when growing in less favorable conditions, since limitations on nutritional availability, or abiotic stresses such as drought, extreme temperatures, or humidity seem to exacerbate any trade-offs between growth and resistance (Ariga *et al*., 2017; Karasov *et al.*, 2017).

### Considerations on the kinetics, amplitude, and duration of NLR induction

While increasing NLR protein levels via PRR signaling after pathogen detection (Navarro *et al*., 2004; Bjornson and Zipfel, 2021; Tian *et al.*, 2021) is obviously relevant, the kinetics of its induction, control of its amplitude, and its timely repression might be as important. In this regard, the miRNA/phasiNLR regulatory module responsive to PRR-mediated pathogen detection (Shivaprasad *et al*., 2012; Boccara *et al*., 2014; Su *et al*., 2018; López-Márquez *et al*., 2021; Vasseur *et al*., 2022, Preprint) may be particularly beneficial.

With regards to induction kinetics, it bypasses the need for mRNA synthesis and processing from *NLR*-encoding genes, and is, therefore, a fast and efficient way to boost NLR production and hence the immune system at large following pathogen attack. The increase in NLR expression licensed by the reduction in the levels of trigger miRNAs has indeed been shown to enhance NLR-dependent quantitative resistance through potentiating PRR-mediated responses in the absence of specific effector-mediated activation of any NLR (Boccara *et al*., 2014; Su *et al*., 2018; López-Márquez *et al*., 2021; Ngou *et al*., 2021; Yuan *et al.*, 2021a). It has also been proposed that the advantage of combined 22 nt miRNA/phasiRNA over direct 21 nt miRNA action in the regulation of NLRs is the possibility to expand the network of NLRs regulated by a single trigger miRNA (Zhai *et al*., 2011; Shivaprasad *et al.*, 2012; Boccara *et al*., 2014; López-Márquez *et al.*, 2021), thereby further enhancing the response amplitude and broadening the range of pathogens targeted.

Because of the potency of NLR signaling to restrict growth and induce cell death, uncontrolled response amplitudes and durations are not desirable. The miRNA/phasiNLR regulatory module may also be beneficial. For example, after pathogen perception, the increased availability of target NLR mRNAs triggered by PRR activation may sustain phasiNLR production even when trigger miRNA levels decrease (López-Márquez *et al.*, 2021). Such a fast, but gradual, increase in NLR mRNA levels may contribute to a controlled deployment of defenses and also may contribute to explain the subtle impact of experimentally altered trigger miRNA levels on defenses, including PTI (Li *et al.*, 2012b; Boccara *et al.*, 2014; López-Márquez *et al.*, 2021; Vasseur *et al.*, 2022, Preprint), RPS5-mediated ETI (Boccara *et al.*, 2014), and ISR (Niu *et al*., 2016; Nie *et al*., 2019; Jiang *et al*., 2020). In such a scenario, miRNA/phasiNLR regulation would not cease immediately upon pathogen attack and could be important to fine-tune NLR levels throughout the plant response against the pathogen. It can also be hypothesized that miRNAs and phasiRNAs may help to quickly bring back levels of NLRs, whether involved in boosting PRR-mediated or effector-triggered NLR-activated immunity, to their steady-state levels once the incoming threat has been controlled, thus resetting the system. Such a reset of mRNA target levels has been previously reported (Liu *et al*., 2014; López-Márquez *et al.*, 2021). Thus, the combined miRNA/phasiNLR system may help avoid potential side effects of a disproportionate immune activation by tuning both the amplitude and the duration of the response correctly. An additional physiological consequence of such control could be to act as a barrier against excessive NLR-dependent cell death, a process exploited by necrotrophic pathogens (Lorang *et al.*, 2012; Pitsili *et al*., 2020).

### Benefits of employing a non-cell-autonomous silencing system

The involvement of phasiNLR in NLR regulation during defense deployment opens up the interesting possibility that differences in movement between miRNAs and siRNAs may imply the combination of cell-autonomous and non-cell-autonomous control of NLR production (Felippes *et al*., 2011). If phasiNLRs share this ability to engage in pronounced non-cell-autonomous action with other amplified siRNAs, it could be important in preventing run-away cell death, helping to keep in check the hypersensitive response trigger upon NLR-activated ETI. In this aspect, phasiRNA could also provide additional benefits by controlling the spatial reach and amplifying the reset silencing process.

### Trigger miRNA/phasiNLR control as a means of RNAi-directed pathogen surveillance

Many pathogens with completely different life styles including viruses, bacteria, oomycetes, and fungi employ effectors capable of suppressing RNA silencing to manipulate host gene expression programs and/or suppress the RNA silencing involved in defense (Anandalakshmi *et al*., 1998; Béclin *et al.*, 1998; Kasschau and Carrington, 1998; Voinnet *et al*., 1999; Navarro *et al*., 2008; Qiao *et al*., 2013; Csorba *et al*., 2015; Hou *et al*., 2019). Therefore, the miRNA/phasiNLR-based regulation of NLRs may constitute a simple mechanism to sense such anti-RNAi effector activities to release NLR expression to switch on the immune response (Li *et al*., 2012b; Shivaprassad *et al*., 2012; Fei *et al*., 2013, 2016). Importantly, pathogens do not just target the miRNA branch of RNAi pathways; some target DICER-LIKE proteins involved in siRNA generation and others target proteins such as SGS3 or RDR6, key components of amplified RNA silencing (Csorba *et al*., 2015). The trigger miRNA/phasiNLR system ensures that many NLR-encoding genes are under the control of at least one branch of RNAi acting at the post-transcriptional level (Axtell, 2013; Bologna and Voinnet, 2014; Borges and Martienssen, 2015). This setting may therefore allow plants to sense any pathogen that uses an effector to target any step in post-transcriptional RNAi, as suggested by Vasseur and collaborators (Vasseur *et al.*, 2022, Preprint). Seen in this way, the trigger miRNA/phasiNLR system may be viewed as a variant guard model for indirect sensing of the broadest possible spectrum of RNAi-targeting pathogen effectors. Instead of conformational changes of a single NLR leading to its activation, this surveillance system would react by increasing NLR expression, thereby probably increasing the likelihood that some NLRs wind up in the active conformation to induce immune responses.

## Open questions for future research

Despite progress, the precise roles, and modes of action of phasiRNAs targeting NLRs are still ill defined. Several relevant aspects of their mode of action will require further research. (i) Are phasiNLR more prone to operate through translational repression or by cleaving the target mRNA? (ii) Can phasiNLRs move in a non-cell-autonomous manner reaching miRNA-free tissues as has been reported for other siRNAs? And if so, is this a relevant aspect on immune regulation? Moreover, there are additional open questions on the field that are relevant for understanding how this mechanism of regulation operates. A deeper search into sRNA libraries (http://ipf.sustech.edu.cn/pub/asrd; Yu *et al*., 2017; Feng *et al*., 2020) unraveled that in Arabidopsis both miR472 and miR825-5p are associated with AGO10 (ZWILLE), the closest paralog of AGO1 that was proposed as a transitivity inducer (Iki *et al*., 2018). However, AGO10 mediates the translational repression of mRNA targets (Brodersen *et al*., 2008; Mallory *et al*., 2009; Martin-Merchan *et al.*, 2023) and competes with AGO1 for miR165/166 binding, inhibiting the function of those miRNAs during apical shoot development and triggering their degradation (Zhu *et al*., 2011; Yu *et al*., 2017). AGO10 also sequesters miR398 and prevents its movement (Cai *et al*., 2021), a result that supports the role of AGO10 as a miRNA locker (Manavella *et al*., 2011). These observations open up relevant questions such as the following. (i) Are these two proteins contributing to phasiNLR biogenesis through miR472 and miR825-5p? (ii) Is the partitioning of those miRNAs onto AGO1 and AGO10 relevant for their mode of action (slicing versus translational repression)? Or may it vary according to AGO1/AGO10 expression patterns? And finally, (iii) is AGO10 acting as a miRNA locker impeding NLR regulation in some specific tissues?

It is also intriguing how the large differences in the number of miRNAs involved in NLR regulation and that of phasiNLR loci existing between different species may affect the mode of action or the possible signaling networks involved in regulating NLR function in these species. Future analysis comparing the NLR networks and functional impacts on such different systems could provide insightful information on both the mechanistic implications and how these differences evolved. In this regard, generation of a few miRNA loss-of-function mutants (knockouts) in selected species via the CRISPR (clustered regularly interspaced short palindromic repeats)/Cas (CRISPR-associated protein) technology would provide valuable information.

Another aspect important for understanding the relevance of this type of regulation in plant defenses that has not been investigated at the molecular level is how NLR-targeting miRNAs and phasiNLRs respond to PRR-mediated pathogen perception. The link between PAMP perception and decreases in transcription of the *MIR* loci and mature miRNA levels is clear; however, the signaling elements involved are still unknown. Additionally, mature miRNA levels have been shown to drop as soon as 30 min after PAMP treatment (Boccara *et al*., 2014; Su *et al.*, 2018; Vasseur *et al*., 2022, Preprint). However, the longer average half-lives of miRNAs in other systems (Marzi *et al*., 2016; Reichholf *et al*., 2019) suggest that plant cells may possess a mechanism to quickly get rid of those miRNAs and boost the immune response. It is tempting to speculate that the appearance (in terms of evolution) of endogenous target mimics or sponges may be an easy explanation for these observations, resembling the case of miR399 and the endogenous target mimic *IPS1* during phosphate starvation (Franco-Zorrilla *et al*., 2007). It is important to note that plant genomes contain a number of non-coding NLR-derived transcripts of uncharacterized function. These transcripts may serve as a template for phasiNLR biogenesis, as is the case for *TAS5* in tomato (Li *et al*., 2012a; Canto-Pastor *et al.*, 2019). However, due to sequence similarities, these long non-coding RNAs may constitute the source RNA molecules from which miRNA/phasiNLR sponges could originate.

*Solanaceae*-specific 22 nt miR6019 triggers the production of phasiNLR from the target NLR N gene conferring resistance against TMV in *Nicotiana tabacum* (Li *et al.*, 2012b). However, miR6019 has been shown to be transcriptionally repressed during plant maturation, a phenomenon accompanied by heightened levels of *N* mRNAs and increased *N*-mediated resistance against TMV in tobacco plants, a mechanism that may be conserved in other *Solanaceace* species (Deng *et al.*, 2018). Thus, this work could be considered the first to establish adjustments of a ‘miRNA–phasiNLR module’ linked to plant development that could be important to fine-tune NLR expression during this process. Additional work on this potential link in other species and regulatory modules may be important to understand the relevance of this type of regulation and to explain its prevalence.

Much have been discovered about the role of 22 nt miRNA and phasiNLR regulation in plant immunity in the last decade. This, in conjunction with the developments in the last few years in defense signaling, make this a hot and exciting topic with a great potential to be unlocked in generating new strategies for crop protection, in which much is yet to be learnt.

## Abbreviations:

CC: coiled-coil
CNL: CC-NLR
ETI: effector-triggered immunity
LRR: leucine-rich repeat
NB: nucleotide binding
NLR: NB-LRR or NOD-like receptor
PTI: PAMP-triggered immunity
TIR: Toll/interleukin-1 receptor
TNL: TIR-NLR
